# Protective Effects of *Gymnema inodorum* Leaf Extract on *Plasmodium berghei*-Induced Hypoglycemia, Dyslipidemia, Liver Damage, and Acute Kidney Injury in Experimental Mice

**DOI:** 10.1155/2021/1896997

**Published:** 2021-09-12

**Authors:** Kongsak Boonyapranai, Sirirat Surinkaew, Voravuth Somsak, Rujikorn Rattanatham

**Affiliations:** ^1^Research Institute for Health Sciences, Chiang Mai University, Chiang Mai 50200, Thailand; ^2^School of Allied Health Sciences, Walailak University, Nakhon Si Thammarat 80160, Thailand; ^3^Research Excellence Center for Innovation and Health Products, Walailak University, Nakhon Si Thammarat 80160, Thailand

## Abstract

Malaria complications are the most frequent cause of mortality from parasite infection. This study is aimed at investigating the protective effect of *Gymnema inodorum* leaf extract (GIE) on hypoglycemia, dyslipidemia, liver damage, and acute kidney injury induced by *Plasmodium berghei* infection in mice. Groups of ICR mice were inoculated with 1 × 10^7^ parasitized erythrocytes of *P. berghei* ANKA and administered orally by gavage with 100, 250, and 500 mg/kg of GIE for 4 consecutive days. Healthy and untreated controls were given distilled water, while the positive control was treated with 10 mg/kg of chloroquine. The results showed that malaria-associated hypoglycemia, dyslipidemia, liver damage, and acute kidney injury were found in the untreated mice as indicated by the significant alteration of biological markers. On the contrary, in 250 and 500 mg/kg of GIE-treated mice, the biological markers were normal compared to healthy controls. The highest protective effect was found at 500 mg/kg similar to the CQ-treated group. However, GIE at a dose of 100 mg/kg did not show protection during malaria infection. This study demonstrated that GIE presented potential therapeutic effects on PbANKA-induced hypoglycemia, dyslipidemia, liver damage, and acute kidney injury. The results obtained confirm the prospect of *G. inodorum* as an essential source of new antimalarial compounds and justify folkloric use as an alternative malarial treatment.

## 1. Introduction

Malaria is one of the main public health problems, and it remains one of the serious diseases in developing countries. It is a parasitic disease caused by protozoa in the genus *Plasmodium* transmitted via the bite of an infected female *Anopheles* mosquito. According to the World Health Organization (WHO), in 2018, they estimated 228 million cases and 405,000 deaths globally, with over 94% of malaria deaths occurring in the African region [1]. Due to the resistance of *Plasmodium* malaria parasites to standard available drugs, the chemotherapy of malaria has become challenging [2]. Additionally, malaria infection also induces the severity of life-threatening complications, including cerebral malaria, metabolic acidosis, severe hemolytic anemia, hypoglycemia, dyslipidemia, lung injury and pulmonary distress, liver damage, and acute kidney injury [3]. Malaria-associated hypoglycemia, dyslipidemia, liver damage, and acute kidney injury are correlated with high mortality in malaria patients. Oxidative stress and proinflammatory cytokine production during malaria propagation are strongly associated with these complications [4, 5]. Hence, a search for new antimalarial drugs for chemotherapy and complementary approaches is urgently needed. Approximately 80% of the world's population relies on traditional medicinal plants for their healthcare. Since artemisinin and quinine originate from plants, other traditional medicinal plants that have a folklore reputation must be explored and determined for their pharmacological potential as sources of alternative antimalarial drugs [6, 7].

*Gymnema sylvestre* (family Asclepiadaceae) is a medicinal plant that is used as a traditional therapy in many regions of India, Asia, and Australia to treat certain diseases such as dyspepsia, constipation, jaundice, hemorrhoids, hepatitis, kidney disease, cardiopathy, asthma, diabetes mellitus, and malaria [8]. Many previous studies have demonstrated multifarious pharmacological potentials, including antioxidant, anti-inflammatory, anticancer, immunosuppressive, hypoglycemic, anti-infectious, hepatoprotective, and nephroprotective activities [9]. These phytochemical activities derived from *G. sylvestre* have been presented, such as polyphenols, flavonoids, alkaloids, triterpenoids, saponins, anthraquinones, and gymnemic acids [10]. *Gymnema inodorum*, also a member of the Asclepiad strain, is found indigenously in Thailand, particularly in the northern region. *G. inodorum* has the potential to inhibit glucose absorption and decrease blood glucose levels [11–13]. Moreover, it was found that *G. inodorum* leaf extract presented high antioxidant activity with polyphenols as significant constituents [14]. However, no scientific evidence on the therapeutic effect of *G. inodorum* has ever been reported in malaria-infected mice. Therefore, this study is aimed at evaluating the protective and therapeutic effects of *G. inodorum* leaf extract on malaria-induced hypoglycemia, dyslipidemia, liver damage, and acute kidney injury in *Plasmodium berghei*-infected mice.

## 2. Materials and Methods

### 2.1. Preparation of Aqueous Crude Extract and Antimalarial Drugs

*Gymnema inodorum* leaves were collected from Chiang Mai, Thailand. This plant was identified and authenticated by the botanist at the Faculty of Sciences, Chiang Mai University. The voucher specimen (NRU64/036-001) was deposited at Chiang Mai University. Dried powdered plant materials were extracted by dissolving in distilled water (DW) at a proportion of 5 g% in the incubator shaker (60°C) for 15 min. Centrifugation at 2,500 rpm for 15 min was carried out, and the supernatant was then collected. The pellet was reextracted, and the supernatant was subsequently combined. Lyophilization was carried out to obtain dried powdered aqueous crude extract (GIE) which was then stored at –20°C for further use [13]. Our previous study revealed that up to 5,000 mg/kg of GIE with no mortality or signs of toxicity was observed. Hence, the optimal doses for administration in mice used in this study were 100, 250, and 500 mg/kg [15].

### 2.2. Mice

Pathogen-free male mouse strain ICR 4-6 weeks old, weighing 25-30 g, purchased from Nomura Siam International, were used in this study. Mice were acclimatized for at least one week before the experiments. They were housed in the standard condition of 12 h light-dark cycle, 22-25°C with free access to a standard pellet diet and clean water *ad libitum*. All experiments associated with animals were ratified and approved by the Animal Ethics Committee, Walailak University, with the EC number of WU-AICUC-63-031.

### 2.3. *Plasmodium berghei*

Chloroquine-sensitive *Plasmodium berghei* strain ANKA used in this study was obtained from the Malaria Research and Reference Reagent Resource Center (MR4). PbANKA in the form of cryopreservative stock was entirely thawed in a water bath at 37°C and subsequently inoculated into naïve ICR mice. Parasitemia was monitored daily by microscopic examination of Giemsa-stained thin blood films. A serial passage from PbANKA-infected donors to naïve mice was performed on a weekly basis. Additionally, biochemical markers were also monitored in triplicate on days 0, 2, 4, 6, 8, and 10 postinfection.

### 2.4. Determination of Hypoglycemia, Liver Damage, and Acute Kidney Injury

Evaluation of hypoglycemia, liver damage, and acute kidney injury induced by PbANKA infection in mice was indicated by biochemical blood markers including blood glucose, total cholesterol, triglycerides, aspartate aminotransferase (AST), alanine aminotransferase (ALT), alkaline phosphatase (ALP), blood urea nitrogen (BUN), and creatinine. Mouse blood was collected by cardiac puncture into a heparinized vacuum tube, and centrifugation at 2,000 g for 10 min was then performed. Plasma was collected and used as a subject for the measurement of biochemical markers using a clinical chemistry automated analyzer with specific reagent kits from Mindray, Shenzhen, China. Blood glucose was measured by the glucose oxidase-peroxidase (GOD-POD) method. Total cholesterol was analyzed by the cholesterol oxidase-peroxidase (CHOD-POD) method, while triglyceride was measured by the glycerokinase peroxidase-peroxidase (GPO-POD) method. AST, ALT, and ALP were measured by UV-assay according to the International Federation of Clinical Chemistry and Laboratory Medicine (IFCC) without pyridoxal phosphate activation. BUN was measured by the urease-glutamate dehydrogenase (GLDH) UV method. Creatinine was analyzed by the modified Jaffe method.

### 2.5. Efficacy Assay of *G. inodorum* in Mice

According to standard Peter's test, the protective efficacy of GIE in mice was carried out as previously described [16]. Naïve ICR mice were divided into 6 groups (3 mice per group) and inoculated with 1 × 10^7^ parasitized erythrocytes of PbANKA by IP injection. Two hours later, these mice were administered by oral gavage of 100, 250, and 500 mg/kg of GIE once a day for 4 consecutive days (days 0-3). The healthy and untreated groups were given 10 ml/kg of DW. In addition, 10 mg/kg of CQ was also used for treatment as a positive control. At the end of the treatment (day 4), mice in all groups were sacrificed, and blood was then collected by cardiac puncture into heparinized vacuum tubes. Parasitemia and biochemical markers were then measured as previously described above. Then, the percent inhibition was estimated by using the formula below:

(1) $$\begin{array}{c} \%\text{inhibition}=\frac{\left(\text{parasitemia of untreated group}–\text{parasitemia of tested group}\right)\times 100}{\text{parasitemia of untreated group}}. \end{array}$$

### 2.6. Mean Survival Time

In this study, mortality was monitored by recording the number of days from the time of infection up to death in each mouse for 30 days. The mean survival time (MST) was subsequently calculated using the formula below:

(2) $$\begin{array}{c} \text{MST}=\frac{\text{sum of survival time of all mice in a group}}{\text{total number of mice in a group}}. \end{array}$$

### 2.7. Statistics

Statistical analysis was performed using GraphPad Prism 6.0 (GraphPad Software, Inc., San Diego, CA). All results of this study were expressed as the mean and standard error of the mean (SEM). The comparison between the control and treatment groups was performed using one-way analysis of variance (ANOVA) and Tukey's posttest. Significant differences were considered at 95% confidence, *p* < 0.05.

## 3. Results

### 3.1. PbANKA-Induced Hypoglycemia, Dyslipidemia, Liver Damage, and Acute Kidney Injury

**Figure 1:**
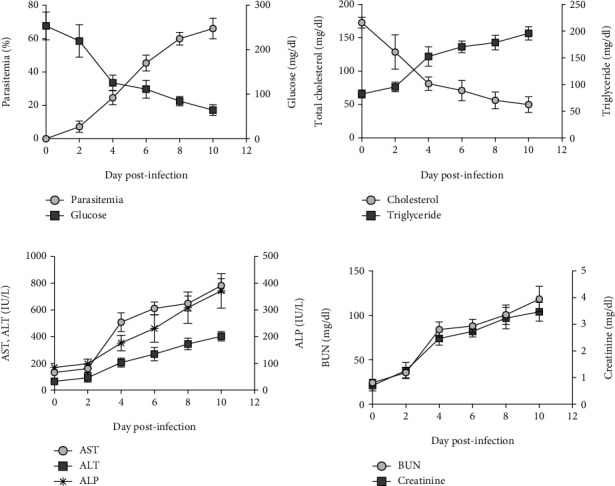
PbANKA-induced hypoglycemia, dyslipidemia, liver damage, and acute kidney injury in mice. ICR mice were inoculated with 1 × 10^7^ parasitized erythrocytes of PbANKA by intraperitoneal injection. (a) Parasitemia and blood glucose, (b) total cholesterol and triglyceride, (c) AST, ALT, and ALP, and (d) BUN and creatinine levels were monitored. Results were expressed as the mean ± SEM.

As shown in Figure 1, hypoglycemia, dyslipidemia, liver damage, and acute kidney injury induced by PbANKA infection in mice were observed. Hypoglycemia during the infection was indicated by a particular decrease in blood glucose levels (Figure 1(a)). We observed that total cholesterol was also decreased with markedly increased levels of triglycerides (Figure 1(b)). Moreover, elevated levels of AST, ALT, and ALP were detected (Figure 1(c)). Additionally, we observed an increase in BUN and creatinine in the infected mice (Figure 1(d)). These findings were consistent with the increased levels of parasitemia in PbANKA-infected mice from day 4 onward.

### 3.2. Effect of GIE on Parasitemia against PbANKA Infection

**Figure 2:**
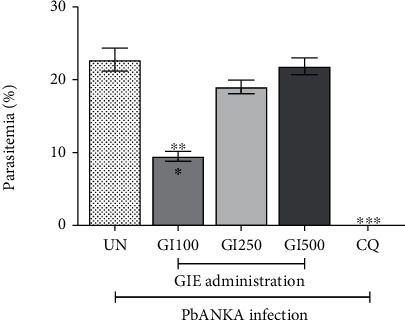
Effect of GIE on parasitemia against PbANKA infection. Groups of ICR mice were inoculated with 1 × 10^7^ parasitized erythrocytes of PbANKA by intraperitoneal injection. GIE at the doses of 100, 250, and 500 mg/kg were administered orally once a day for 4 consecutive days. CQ (10 mg/kg) was given as positive control. Parasitemia was estimated. ^∗∗∗^*p* < 0.001, compared to UN. UN: untreated mice; GI100, GI250, and GI500: treated mice with 100, 250, and 500 mg/kg of GIE; CQ: 10 mg/kg of chloroquine-treated mice. Results were expressed as the mean ± SEM.

The antimalarial activity of GIE against PbANKA infection is shown in Figure 2. Only GIE at a dose of 100 mg/kg showed a significant (*p* < 0.01) reduction in parasitemia compared to the untreated group with a percent inhibition of 58.28%. However, 250 and 500 mg/kg of GIE did not show an antimalarial effect against PbANKA. In addition, CQ decreased the parasitemia level to undetectable.

### 3.3. Effect of GIE on Blood Glucose, Total Cholesterol, and Triglycerides during PbANKA Infection

**Figure 3:**
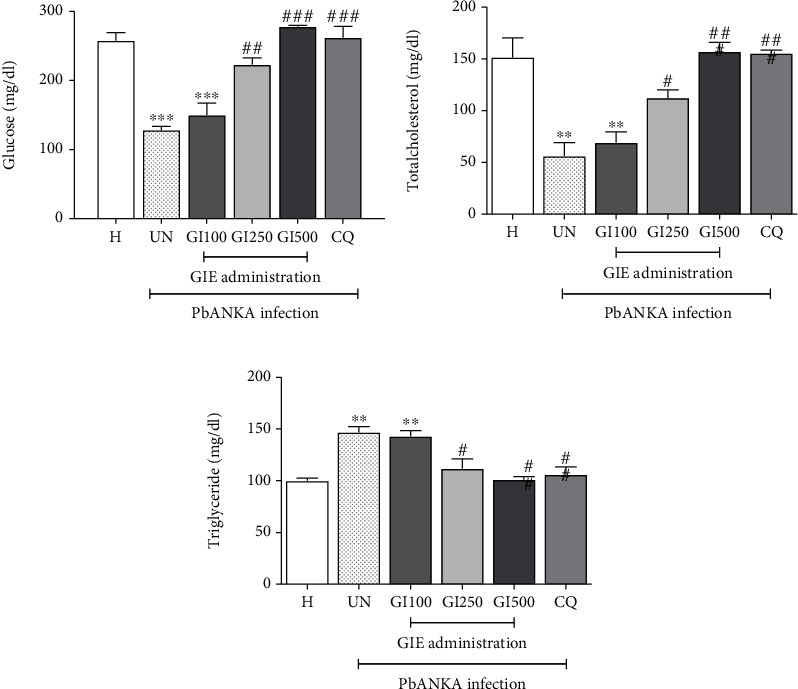
Effect of GIE on blood glucose, total cholesterol, and triglycerides during PbANKA infection. Groups of ICR mice were inoculated with 1 × 10^7^ parasitized erythrocytes of PbANKA by intraperitoneal injection. GIE at the doses of 100, 250, and 500 mg/kg were administered orally once a day for 4 consecutive days. CQ (10 mg/kg) was given as the positive control. Blood was collected for measurement of (a) blood glucose, (b) total cholesterol, and (c) triglyceride. ^∗∗^*p* < 0.01 and ^∗∗∗^*p* < 0.001, compared to H. ^#^*p* < 0.05, ^##^*p* < 0.01, and ^###^*p* < 0.001, compared to UN. H: healthy mice; UN: untreated mice; GI100, GI250, and GI500: treated mice with 100, 250, and 500 mg/kg of GIE; CQ: 10 mg/kg of chloroquine-treated mice. Results were expressed as the mean ± SEM.

Figures 3(a)–3(c) (UN) show that significantly (*p* < 0.01) decreased blood glucose and total cholesterol with increasing triglyceride levels occurred in untreated mice over the 4 days of the study, compared to healthy controls. The normalization of blood glucose, total cholesterol, and triglyceride levels during PbANKA infection was found in GIE-treated groups at the doses of 250 and 500 mg/kg but not in 100 mg/kg (Figures 3(a)–3(c); GI100, GI250, and GI500). 500 mg/kg of GIE presented the highest efficacy similar to CQ-treated mice.

### 3.4. Effect of GIE on AST, ALT, and ALP during PbANKA Infection

**Figure 4:**
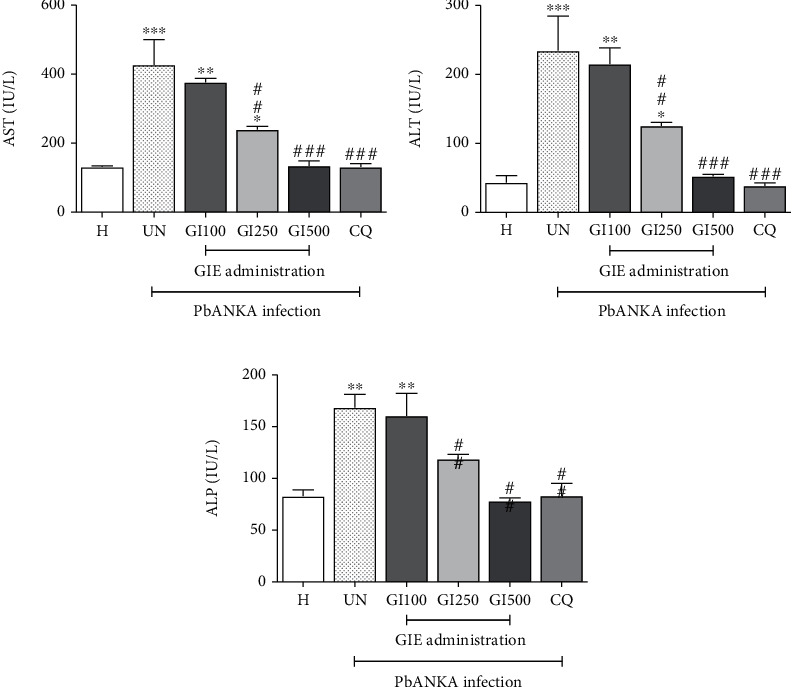
Effect of GIE on AST, ALT, and ALP during PbANKA infection. Groups of ICR mice were inoculated with 1 × 10^7^ parasitized erythrocytes of PbANKA by intraperitoneal injection. GIE at the doses of 100, 250, and 500 mg/kg were administered orally once a day for 4 consecutive days. CQ (10 mg/kg) was given as positive control. Blood was collected for measurement of (a) AST, (b) ALT, and (c) ALP. ^∗^*p* < 0.01, ^∗∗^*p* < 0.01, and ^∗∗∗^*p* < 0.001, compared to H. ^##^*p* < 0.01 and ^###^*p* < 0.001, compared to UN. H: healthy mice; UN: untreated mice; GI100, GI250, and GI500: treated mice with 100, 250, and 500 mg/kg of GIE; CQ: 10 mg/kg of chloroquine-treated mice. Results were expressed as the mean ± SEM.

Significantly (*p* < 0.01) elevated liver enzymes AST, ALT, and ALP were observed in the untreated group, compared to the healthy control (Figures 4(a)–4(c); UN). PbANKA-infected mice treated with 250 and 500 mg/kg of GIE showed hepatoprotection as indicated by normal levels of these enzymes, compared to healthy mice (Figures 4(a)–4(c); GI250, GI500). However, the 100 mg/kg GIE-treated group did not protect against liver damage (Figures 4(a)–4(c); GI100). The highest efficacy was found in 500 mg/kg GIE-treated mice.

### 3.5. Effect of GIE on BUN and Creatinine during PbANKA Infection

**Figure 5:**
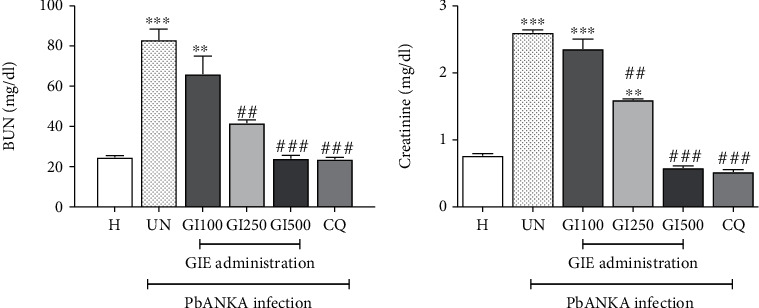
Effect of GIE on BUN and creatinine during PbANKA infection. Groups of ICR mice were inoculated with 1 × 10^7^ parasitized erythrocytes of PbANKA by intraperitoneal injection. GIE at the doses of 100, 250, and 500 mg/kg were administered orally once a day for 4 consecutive days. CQ (10 mg/kg) was given as positive control. Blood was collected for measurement of (a) BUN and (b) creatinine. ^∗∗^*p* < 0.01 and ^∗∗∗^*p* < 0.001, compared to H. ^##^*p* < 0.01 and ^###^*p* < 0.001, compared to UN. H: healthy mice; UN: untreated mice; GI100, GI250, and GI500: treated mice with 100, 250, and 500 mg/kg of GIE; CQ: 10 mg/kg of chloroquine-treated mice. Results were expressed as the mean ± SEM.

As shown in Figures 5(a) and 5(b) (UN), PbANKA infection induced acute kidney injury as indicated by significantly (*p* < 0.01) increased levels of BUN and creatinine. The infected mice administered orally with GIE (250 and 500 mg/kg) showed normal BUN and creatinine levels but not in 100 mg/kg (Figures 5(a) and 5(b); GI100, GI250, and GI500). The highest protective effect on acute kidney injury was observed in 500 mg/kg of GIE, similar to CQ-treated mice.

### 3.6. Effect of GIE on Survival Time of Mice

In standard Peter's test, the 100, 250, and 500 mg/kg GIE-treated groups were correlated with significantly (*p* < 0.01) increased MST compared to the untreated groups in a dose-dependent manner (Table 1).

## 4. Discussion

In this study, we carried out experiments to investigate the protective effects of the extract from the leaves of *G. inodorum* on PbANKA-induced hypoglycemia, dyslipidemia, liver damage, and acute kidney injury in mice, regardless of whether it had an antimalarial effect. As indicated by the marked decrease in blood glucose concentration, the results showed that hypoglycemia was observed in PbANKA-infected mice, consistent with previous studies [17]. This could be due to the fact that hypoglycemia is a frequently encountered complication during malaria infection that is usually ascribed to increased glucose utilization by parasitized erythrocytes, malabsorption of glucose, increased tissue metabolism, and impaired glucose production via the inhibition of gluconeogenesis [18, 19]. *Plasmodium* has no capacity to store glucose as an energy source in the form of glycogen; they entirely rely on an exogenous glucose supply. It has been reported that the parasitized erythrocytes exhibited an increased permeability for glucose through the facilitated hexose transporter, and subsequently, the metabolism of the parasite is utilized through the process of glycolysis [20]. This is accompanied by approximately 75-100 times more glucose utilization than normal erythrocytes, thus causing hypoglycemia if untreated [21]. The liver is one of the major sites of metabolic processes and is the main regulator of glucose and lipid metabolism [22]. Alterations of glucose and lipid metabolism during severe malaria infection have been described [23, 24]. Perturbation in hepatic glucose metabolism in murine progression of malaria has also been reported [25], and hypoglycemia was relevant to liver damage [26]. Liver damage was confirmed in PbANKA-infected mice as suggested by the significant increase of liver enzymes AST, ALT, and ALP, consistent with previous studies [27]. Several studies suggested that parasitized erythrocyte sequestration contributed to liver obstruction of blood flow followed by hypoxia and excessive hemolysis, causing increased oxidative stress and leukocyte infiltration [28]. Moreover, inflammatory cytokines produced by leukocytes enhanced liver inflammation and cell damage [27]. In the model used for this study, hemozoin, a free heme derived from high parasitemia and hemolysis, was likely to contribute to the oxidative stress response in the liver [29]. Another study demonstrated that PbANKA infection caused hydropic degeneration, and continuous degeneration can cause liver cell necrosis [30].

The results obtained in this study showed dyslipidemia in mice infected with PbANKA; triglyceride was found significantly higher in malaria infection. Elevated triglyceride is also a characteristic of liver damage [22]. Malaria infection resulted in the altered expression of genes related to lipid metabolism. The parasite infection causes downregulation of the enzyme 5′ AMP-activated protein kinase (AMPK) and subsequent activation pathways, leading to increased lipid synthesis [31]. Furthermore, a clear relationship has been described between high triglyceride levels and anemia [32]. One hypothesis is that triglyceride is derived from the phospholipids released by the erythrocyte membrane following malaria-induced hemolysis [33]. Moreover, oxidative stress during infection induced lipid peroxidation, which is deleterious for the erythrocyte membrane [34]. A significant decrease was found in the total cholesterol during the infection. This could probably be due to a corresponding decrease in blood glucose in PbANKA-infected mice. During the replicative stages of the malarial parasite, it has been observed to have a tremendous requirement of cholesterol for the intracellular parasite development and the maintenance of the trophozoite stage [35, 36]. The mature schizonts are composed of 8-32 merozoites that require cholesterol for the maintenance of their infectivity, resulting in hypocholesterolemia [37].

PbANKA infection in mice is a well-known model of malaria-induced acute kidney injury [30]. Here, PbANKA-infected mice showed kidney injury as confirmed through manifestations of significantly increased BUN and creatinine levels in the untreated group, consistent with previous reports [38]. In severe malaria, hypovolemia was developed, leading to the activation of vasoactive mediators, which could be involved in the pathogenesis of malaria-induced acute kidney injury [39]. It has been proposed that PbANKA-induced acute kidney injury depends on parasitized erythrocyte adhesion to renal endothelial cells and exacerbated immune response to oxidative stress products, leading to glomerular and tubular injuries [40, 41]. In addition, lipid peroxidation mediated by heme-derived oxidative stress during PbANKA infection was also considered a major role in malaria-induced acute kidney injury [42]. In response to liver and kidney injury, molecular and cellular reaction cascade would be generated with the aims of restriction damage, regeneration of damaged cells and tissues, and resistance to further infection [43, 44], together with a medicinal plant that may alleviate oxidative damage and modulate the inflammation process in serious conditions. In the present study, GIE-treated mice infected with PbANKA showed glucose homeostasis, restored total cholesterol and triglyceride levels, and abolished liver damage and acute kidney injury. The dose of 500 mg/kg GIE was the most effective compared with the doses of 100 and 250 mg/kg. There was a possibility that apart from polyphenols and other compounds in GIE such as flavonoids, alkaloids, triterpenoids, saponins, quercetins, and gymnemic acids, they may have resulted individually or synergistically in the observed antihypoglycemic, antidyslipidemia, hepatoprotective, and nephroprotective activities of GIE treatment in the present study [10, 14, 45]. This extract might stimulate some antioxidant enzymes involved in the fight against oxidative stress, causing less damage to the host. The recent finding showed the antioxidant properties of GIE by regulating the reactive oxygen species production and activating the antioxidant gene, superoxide dismutase 2 expression, which leads to the suppression of proinflammatory cytokines, interleukin 6 (IL-6), and production and downregulation of inflammatory genes such as cyclooxygenase-2, inducible nitric oxide synthase, and IL-6 mRNA levels in murine macrophages [14]. Consistent with the reports on the pharmacological properties of *G. sylvestre*, gymnemic acid might play a crucial role in the protective effects through antioxidant and anti-inflammatory activities [46]. It is noteworthy that GIE treatment did not display comparable suppressive antimalarial activity to PbANKA even at the highest dose treatment (500 mg/kg). This at least undetectable antimalarial activity of GIE may indicate that the active compounds extracted by this solvent might have less potent antimalarial properties. However, it indicated that the observed protective benefits in this study did not result from the less severe parasitemia in mice treated with GIE. The finding in this study is preliminary. Thus, confirmatory studies followed by identifying the active compounds of GIE that are responsible for the observed protective effects on hypoglycemia, dyslipidemia, liver damage, and acute kidney injury in PbANKA infection in mice are recommended.

## 5. Conclusion

The protective properties of GIE against PbANKA-induced hypoglycemia, dyslipidemia, liver damage, and acute kidney injury in mice without antimalarial activity or toxicity have been demonstrated in this study. The significant recovery of biological markers in the treated mice justifies the folkloric use of *G. inodorum* leaves in malarial treatment.

## Figures and Tables

**Table 1:** Effect of *G. inodorum* leaf extract on MST in PbANKA-infected mice.

Groups	Doses	MST (days)
Untreated mice	DW 10 ml/kg	8.33 ± 0.88
GIE-treated mice	100 mg/kg 250 mg/kg 500 mg/kg	17.33 ± 1.45^∗∗^ 21.67 ± 0.88^∗∗∗^ 26.00 ± 1.15^∗∗∗^
CQ-treated mice	10 mg/kg	30.00 ± 0.00^∗∗∗^

Results were expressed as the mean ± SEM (*n* = 3). ^∗∗^*p* < 0.01 and ^∗∗∗^*p* < 0.001, compared to untreated mice.

## Data Availability

Data is available at https://figshare.com/s/6efb18b84b5e9ab718ae/, https://10.0.23.196/m9.figshare.14316134.
